# *Minneola tangelo* essential oil exhibits antibacterial activity against multidrug-resistant pathogens while maintaining cell safety

**DOI:** 10.1186/s12906-025-05015-5

**Published:** 2025-07-22

**Authors:** Nouran M. Fahmy, Haidy A. Gad, Masarra M. Sakr, Mai I. Shahin, Shaimaa Fayez

**Affiliations:** 1 Department of Pharmacognosy, Faculty of Pharmacy, Ain Shams University, African Union Organization St. Abbassia, Cairo, 11566 Egypt; 2 Department of Pharmacognosy, Faculty of Pharmacy, King Salman International University, South Sinai, 46612 Egypt; 3 Department of Microbiology and Immunology, Faculty of Pharmacy, Ain Shams University, African Union Organization St. Abbassia, Cairo, 11566 Egypt; 4 Department of Pharmaceutical Chemistry, Faculty of Pharmacy, Ain Shams University, African Union Organization St. Abbassia, Cairo, 11566 Egypt

**Keywords:** *Minneola Tangelo* essential oil, GC-MS, Antimicrobial resistance, *in-silico* studies, MRSA, *Acinetobacter baumannii*

## Abstract

**Background:**

The significant rise in antibiotic resistance has become an alarming situation urging the search for new antibacterial agents. Nature has always been a limitless source of bioactives with high safety profile. This study evaluates the antibacterial activity of essential oils from the leaves and fruit peels of *Minneola tangelo* cultivated in Egypt. In vitro cytotoxicity assay was conducted to ensure the safety profile of the active essential oils.

**Methods:**

The antibacterial activity of clinical isolates of *Acinetobacter baumannii* and methicillin-resistant *Staphylococcus aureus* was assessed using the Kirby-Bauer disk diffusion method. Chemical profiling of the leaf and peel essential oils was performed using GC-MS. In vitro cytotoxicity assay of the leaf essential oil was conducted using sulforhodamine B assay. In silico docking study was conducted to explore the possible antibacterial mechanisms.

**Results:**

The leaf essential oil exhibited antibacterial activity against the tested isolates, whereas the peel oil was inactive. GC-MS analysis showed differences in the chemical composition of the leaf and fruit peel oils, where 60% of the leaf oil is dominated by linalool (31.6%), *cis*-β-ocimene (16.1%), and γ-terpinene (14.3%), whereas the fruit peel oil is solely dominated by D-limonene (82%). Cytotoxicity assay on Caco-2 cell line showed IC_50_ value of 277.36 µg/mL while that on fibroblast HFB4 cell line was > 1000 µg/mL. In silico studies revealed high affinity of linalool to FabI, a crucial enzyme in the fatty acid biosynthesis pathway of MRSA as well as an affinity to Penicillin binding protein PBP2a. Binding of linalool to shikimate kinase of *Acinetobacter baumannii* was also demonstrated.

**Conclusion:**

Essential oil of *M. tangelo* is a promising antibacterial agent against multidrug resistant strains with a high safety profile.

**Graphical abstract:**

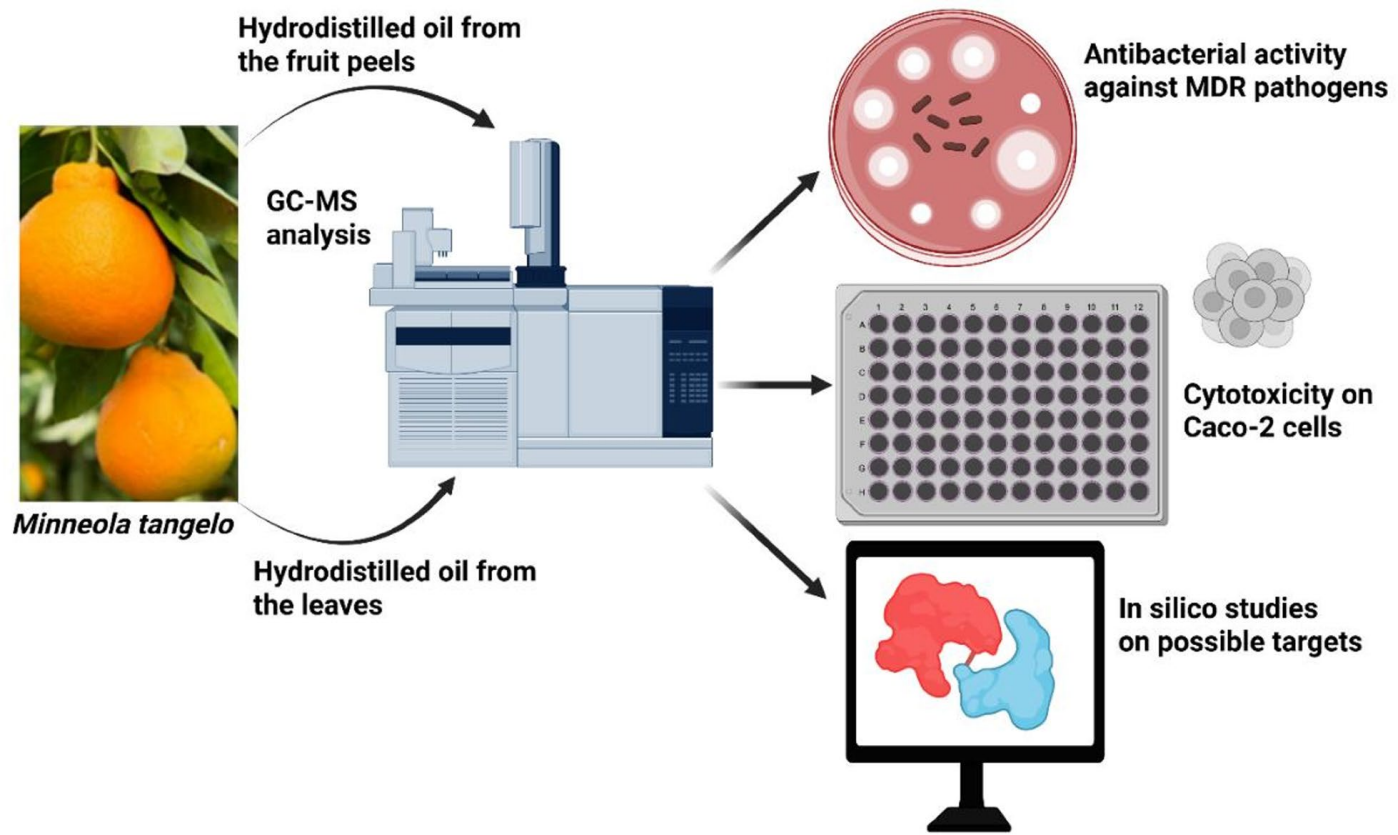

**Supplementary Information:**

The online version contains supplementary material available at 10.1186/s12906-025-05015-5.

## Background

Antibiotics, as the backbone of modern clinical medicine, are facing serious threats from emerging antimicrobial-resistance (AMR) in several pathogenic bacteria posing serious human-health concerns. The World Health Organization, which has previously published a priority pathogens list [1], has recently established a World AMR awareness week (WAAW) in which it was announced that health care in the Eastern Mediterranean region is threatened by antibiotic resistance and multidrug resistant infections calling for an urgent need to address this problem [2]. In 2019, AMR was estimated to directly cause the death of about 1.27 million people and contribute to nearly 5 million deaths where low- and middle-income countries are mostly affected [3]. Organisms of particular concern are *Acinetobacter baumannii* and methicillin-resistant *Staphylococcus aureus* for which alarming resistance rates have been reported [3–6]. *A. baumannii* infections account for about 9% of Gram-negative bacterial infections in ICU patients in Europe and the United States [5]. Infections with MRSA were found to be associated with increased morbidity and mortality [6]. In Egypt, death caused by AMR in 2019 ranked the third, bypassing diabetes, kidney diseases, transport injuries, chronic respiratory diseases, and neurological disorders. Reports stated that “there are five pathogens to be aware of in Egypt”, among which are the Gram-positive *Staphylococcus aureus* and the Gram-negative *Acinetobacter baumannii* [7]. Interestingly, MRSA is not only found in hospitals but also in the community (community-associated MRSA). This situation of increasing antibiotic resistance has led to the announcement of its first ever “National action plan for AMR” [8] and has made the search for an alternative to antibiotics an urgent need.

Plant extracts are rich in secondary metabolites with various biological activities, and they represent a promising source for novel antimicrobial and resistance-modifying compounds [4]. Phytochemicals acting in different mechanisms can be used either alone or in combination with antibiotics to combat resistance. Furthermore, some of these compounds possess cytotoxic activity with good safety profile to be potentially developed into antimicrobial as well as anticancer drugs.

In silico studies have also recently played an important role in combating microbial resistance through uncovering and exploring possible mechanisms and drug targets for compounds with antimicrobial activities [9].

*M. tangelo* -present in the Egyptian market- is a hybrid between tangerine and grapefruit making it an interesting target for research. In this study, and for the first time, components of the Egyptian Tangelo essential oil extracted from the leaves and fruit peels were assessed for their antibacterial activities and compared with respect to their chemical composition. Furthermore, the cytotoxicity of the leaves oil was tested against both cancer and normal cell lines. Molecular docking was then conducted to explain the possible antimicrobial mechanism of the leaf essential oil.

## Materials and methods

### Chemicals and media

Mueller Hinton agar (MHA) was purchased from HiMedia, USA. RPMI 1640 and DMEM media were purchased from Merck, Germany. Antibiotic discs were purchased from Bioanalyse, Turkey. Fetal bovine serum was a product of Gibco, UK.

### Bacterial isolates

Clinical isolates of methicillin-resistant *Staphylococcus aureus* (MRSA) and *Acinetobacter baumannii* were recovered from discharged clinical specimens of Microbiology lab of Al Demerdash hospital, Cairo, Egypt- after obtaining the approval of Research Ethics Committee of Faculty of Pharmacy, Ain Shams University (ACU-FP-RHDIRB2020110301 REC #161). The collected MRSA and *Acinetobacter baumannii* isolates were identified biochemically using the VITEK 2 system (bioMérieux, France), and coded as MRSA 1, 2, 3, 4 and Acin 1, 2, and 3, respectively.

### Antimicrobial susceptibility profiling of the clinical isolates

The Kirby-Bauer disk diffusion method was used to determine the antimicrobial susceptibility of the tested isolates. It was conducted according to the guidelines of the Clinical and Laboratory Standards Institute [10]. Inoculum preparation was first done by suspending freshly isolated colonies (18 to 24 h incubation period) of the test isolates, grown on MHA, in isotonic saline and adjusting the turbidity using 0.5 McFarland standard suspension. Antibiotics belonging to different classes were selected according to the CLSI guidelines. The diameters of the inhibition zones formed -if any- were measured and compared to standard tables. According to these tables, the susceptibility of the isolates was recorded as susceptible (S), intermediate (I), or resistant (R).

### Plant material and essential oil extraction

Fresh leaves and fruits of *M. tangelo* were collected in January 2023 from the Agriculture Research Center, Giza, Egypt. The collected samples were identified and authenticated by Prof. Gamal Farag, Head of Citrus Department. Voucher specimens were kept at the Pharmacognosy Department Herbarium, Faculty of Pharmacy, Ain Shams University with codes (PHG-P-MT-401). Four hundred grams of fresh leaves and peel were separately subjected to hydro-distillation using Clevenger-type apparatus for four hours. Essential oils collected were dried over anhydrous sodium sulfate to remove any moisture content, weighed, stored in amber glass containers, and kept at -20 °C until further use [11]. The yield was calculated based on the initial plant weight (%w/w).

### Antibacterial activity of the extracted oils

Preparation of the inoculum was done by agar diffusion method according to the CLSI 2020 guidelines [10] and conducted as previously described [12]. Freshly isolated colonies (grown overnight on MHA) were suspended in isotonic saline and turbidity was adjusted using 0.5 McFarland standard suspension. Surface inoculation was done on MHA plates and wells were then punched into the agar and filled with the essential oil diluted in DMSO and sterile water at concentrations of 50, 25, 10, and 5 mg/mL. The inhibition zones formed after overnight incubation at 37 °C were then measured and plotted against log concentration to calculate the minimum inhibitory concentration (MIC). The experiment was done in triplicates, and the mean and standard deviations were calculated.

### GC-MS analysis of the tangelo-extracted leaf and fruit peel essential oils

Chemical analysis of the leaf and fruit peel essential oils was done using gas chromatography coupled to mass spectrometry (Shimadzu GCMS-QP 2010, Koyoto, Japan). The gas chromatography was equipped with a DB-5 column (30 m × 0.25 mm × 0.25 μm, Restek, USA). Helium was selected as the carrier gas at a flow rate of 1.41 mL/min. Samples (0.5% v/v) were injected with an injection volume of 1 µL and split ratio 1:15. Mass analysis was done by electron ionization (EI) at 70 eV and analyzed in the scan mode over the range of 35 to 500 amu. Ion source temperature was set at 220 °C and the interface temperature at 280 °C. Compounds identifications were based on the comparison of the mass spectrum of each compound with NIST library online database and NIST-17 library installed on the instrument software. In addition, retention indices were calculated relative to *n*-alkanes C_8_–C_30_ injected under the same conditions. The retention index of each compound was matched with that present in NIST chemistry webbook online library and those previously reported retention indices in the literature.

### Cytotoxicity assay

#### Cells and cell lines

Colon cancer Caco-2 cell line was used to test the cytotoxicity of the leaf essential oil whereas fibroblast (HFB4) cell line was used to test the efficacy of leaf oil on normal cells to determine its safety. Caco-2 cells were maintained in RPMI 1640 media whereas fibroblast HFB4 cells were maintained in DMEM media, both supplemented with 100 µg/mL streptomycin, 100 units/mL penicillin and 10% heat-inactivated fetal bovine serum in a humidified, 5% (v/v) CO_2_ atmosphere at 37 ºC.

#### Cell viability assay using sulforhodamine B (SRB)

Cell seeding was done at a density of 2000 cells/ well in 96-well plates. Cells were exposed to different treatments for 72 h during which five different oil dilutions were tested (10000, 1000, 100, 10, and 1 µg/mL). Cytotoxicity was assessed at the end of exposure using SRB assay as previously described [13]. Absorbance was measured at 545 nm using microplate reader (BioTek instruments, Vermont, USA). Doxorubicin was used as a control. Results were expressed as the relative percentage of absorbance compared to control. Experiments were done in triplicates. Half-maximal inhibitory concentration (IC_50_), the drug concentration at which 50% growth inhibition is achieved, was calculated using GraphPad Prism software, version 5.00 (GraphPad Software, Inc. La Jolla, CA, USA). Cells were also examined using inverted microscope.

### Molecular docking studies

In a trial to explore the mechanism of action of the major *M. tangelo* essential oil constituents, a comprehensive molecular docking study was conducted on different molecular targets known to be expressed in the two tested microorganisms: MRSA and *A. baumannii.* Targeted proteins were retrieved from the Protein Data Bank (PDB) where the water molecules were deleted.

The molecular docking of the compounds along with the co-crystallized ligands were accomplished using the x-ray crystal structures of the designated molecular targets (enoyl-acyl carrier protein reductase FabI (PDB ID: 1QG6), Penicillin binding protein PBP2a (PDB ID: 1MWT), tyrosyl-tRNA synthetase (PDB ID: 1JIJ), topoisomerase II DNA gyrase (PDB ID: 2XCT), carbapenemase (PDB ID: 5WI3), *A. baumannii* DNA gyrase (PDB ID: 7PQM) and shikimate kinase (PDB ID: 4Y0A) which were retrieved from the RCSB protein data bank. Preparation of the proteins’ structures was accomplished through the standard protein preparation protocol of Accelry’s discovery studio 2.5. The hydrogen atoms were affixed, the missing loops were furnished, and the force field parameters were applied using CHARMm. Steepest descent minimization algorithm was used to minimize the structures. The active site was evacuated from the co-crystallized ligands. Our targeted structures were prepared using the prepared ligands protocol for hydrogens to be added and minimization was performed. The output prepared proteins were defined as the receptors and the active site was identified based on the co-crystallized ligand. Docking was conducted using CDOCKER protocol and the ten resulting docking poses were generated for each compound. Both the binding modes and the interactions within the active sites were visualized and scrutinized.–CDOCKER INTERACTION ENERGY was used to assess the affinity of the compounds and to compare with the co-crystallized ligand. 2D and 3D visualization were attained through Discovery studio. RMSD values of the co-crystallized ligands aligned with the corresponding re-docked ones were calculated in order to validate the docking results.

## Results

### Antibiogram analysis of the tested isolates

Results in Table S1 showed that all the collected isolates were multidrug resistant strains exhibiting resistance to nearly all tested antibiotics. *A. baumannii* isolates were susceptible only to vancomycin, while the two MRSA isolates showed sensitivity exclusively to linezolid.

### Antibacterial activity of the essential oils

While the essential oil obtained from the peel exhibited no activity against any of the tested isolates, the leaf essential oil demonstrated varying degrees of antibacterial activity against all isolates. Table 1 presents the MIC values of the leaf essential oil against the tested strains.

**Table 1:** The calculated minimum inhibitory concentration of the essential oil of *M. tangelo* leaf against the tested isolates

Isolate code	MIC (mg/mL) ± SD of the leaf essential oil
MRSA 1	7.58 ± 1.2
MRSA 2	8.2 ± 0.8
MRSA 3	7.6 ± 1.7
MRSA 4	6.17 ± 1.1
Acin 1	15.1 ± 0.6
Acin 2	8.03 ± 1.2
Acin 3	9.7 ± 1.8

### GC-MS-assisted profiling of the essential oils of the leaves and fruit peels of *M. tangelo* cultivated in Egypt

**Fig. 1:**
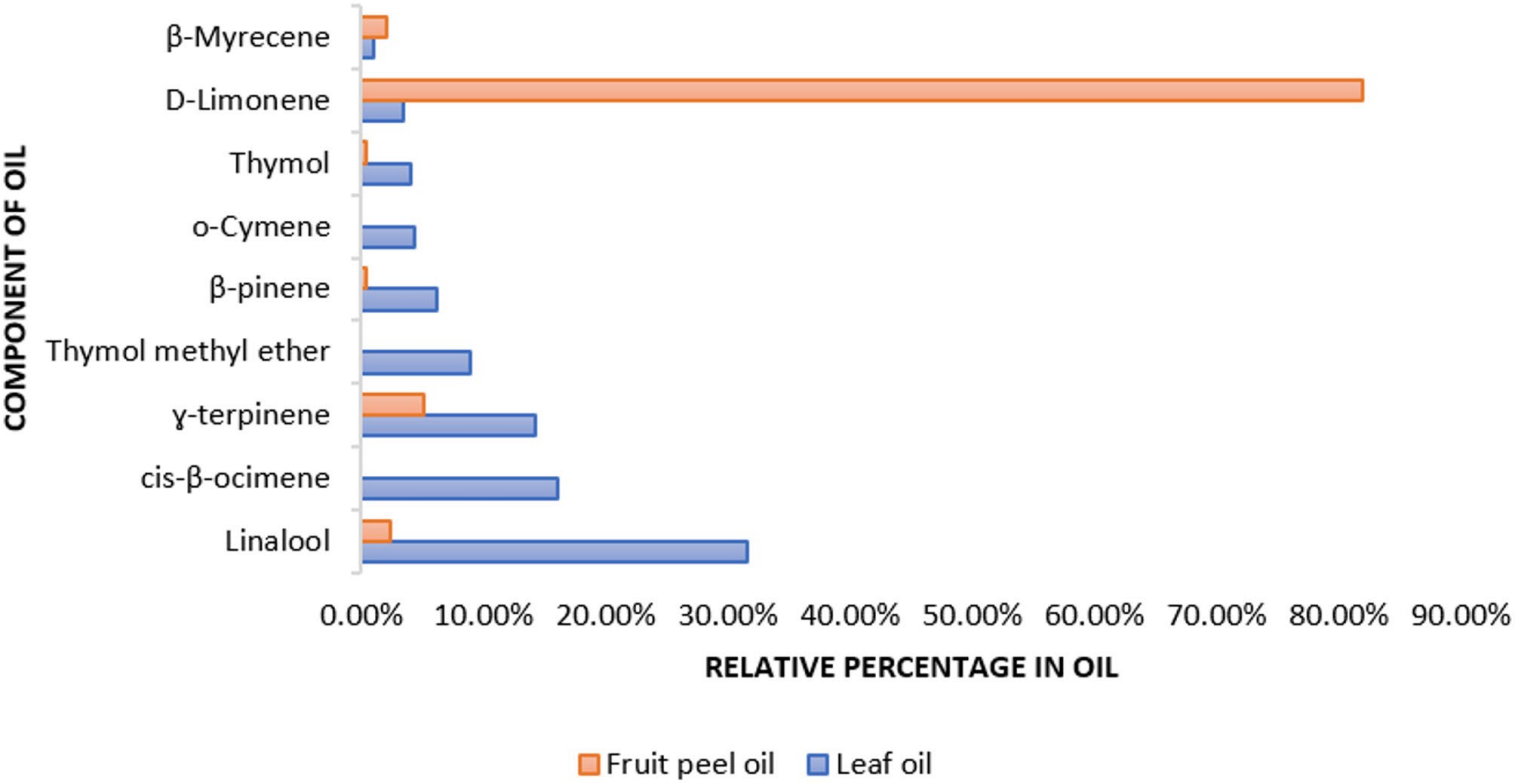
Relative percentage of the major constituents in the essential oils of *M. tangelo* leaves and fruit peels

The essential oil yield varied between the leaves and fruit peels (Fig. 1), with the leaves producing 0.25% oil and the peels yielding 0.57%. GC-MS analysis identified 29 components, accounting for 99.88% of the total leaf oil and 98.76% of the total peel oil. Both oils were predominantly composed of monoterpene hydrocarbons, which made up approximately 53.6% of the leaf oil and 91.3% of the peel oil. Oxygenated terpenes were the second major class, constituting 32.24% of the leaf oil and only 4.43% of the peel oil. Phenolic compounds, particularly thymol and its methyl ether, were detected in considerable amounts in the leaf oil (around 13% of the total oil content) but were present only in trace amounts (0.45%) in the peel oil. GC-MS analysis revealed that linalool was the dominant component in leaf oil, comprising 31.6% of its total content. Other major constituents included cis-β-ocimene (16.1%), γ-terpinene (14.3%), thymol methyl ether (8.9%), β-pinene (6.2%), *o*-cymene (4.3%), thymol (4.0%), and D-limonene (3.5%) (Table 2). In contrast, the fruit peel oil showed up to 82% predominance of D-limonene followed by γ-terpinene (5.13%).

**Table 2:** GC-MS analysis of the essential oils obtained from the leaves and fruit peels of *M. tangelo*

Peak no.	t_*R*_ (min)	Identified compound	Molecular formula	Relative area %		KI_exp_^a^	KI_rep_^b^	Class
				Leaf	Fruit peel			
1.	6.9	*α*-Thujene	C_10_H_16_	1.30	0.18	926	926	Monoterpene hydrocarbons
2.	7.1	*α*-Pinene		2.83	1.05	932	932	
3.	8.3	Sabinene		-	0.15	972	972	
4.	8.4	*β*-Pinene		6.23	0.41	975	975	
5.	8.9	*β*-Myrcene		0.98	2.00	991	991	
6.	9.3	*n*-Capryl aldehyde	C_8_H_16_O	-	0.56	1004	1004	Fatty aldehyde
7.	9.6	4-Carene	C_10_H_16_	0.26	0.13	1016	1014	Monoterpene hydrocarbon
8.	9.9	*o-*Cymene	C_10_H_14_	4.30	-	1024	1024	
9.	10.0	_D_-Limonene	C_10_H_16_	3.59	82.07	1028	1027	
10.	10.3	*trans*-*β*-Ocimene		0.51	-	1038	1031	
11.	10.7	*cis*-*β*-Ocimene		16.11	-	1050	1050	
12.	11.0	γ-Terpinene		14.35	5.13	1060	1058	
13.	11.9	Isoterpinolene		2.97	0.24	1088	1082	
14.	12.3	Linalool	C_10_H_18_O	31.63	2.43	1104	1104	Oxygenated monoterpene
15.	13.5	1,3,8-*p*-Menthatriene	C_10_H_14_	0.25	-	1141	1138	Monoterpene hydrocarbon
16.	14.6	Terpinen-4-ol	C_10_H_18_O	0.32	0.37	1178	1171	Oxygenated monoterpene
17.	15.0	*α*-Terpineol		0.20	1.29	1192	1192	
18.	16.2	*cis*-Myrtanol		-	0.34	1230	1234	
19.	16.4	Thymol methyl ether	C_11_H_16_O	8.99	-	1237	1237	Phenolic compounds
20.	18.1	Thymol	C_10_H_14_O	4.03	0.45	1295	1288	
21.	20.8	*β*-Elemene	C_15_H_24_	0.42	-	1394	1394	Sesquiterpene hydrocarbons
22.	21.6	Caryophyllene		0.24	0.79	1424	1424	
23.	21.7	Aromandendrene		-	0.21	1444	1441	
24.	22.5	*α*-Caryophyllene		0.07	0.10	1458	1458	
25.	23.9	Farnesene		0.14	-	1510	1508	
26.	23.4	*β*-Selinene		-	0.38	1492	1492	
27.	24.1	*α*-Selinene		-	0.48	1500	1501	
28.	25.7	Spathulenol		0.09	-	1584	1584	Oxygenated sesquiterpene
29.	37.0	3,7,11,15-Tetramethyl-2-hexadecen-1-ol		0.10	-	2114	2114	Fatty alcohol
% identified compounds				99.88%	98.76%			

^a^ Kovats index determined experimentally on RTX-5 column relative to C8–C30 *n*-alkanes

^b^ Published Kovats retention indices

Identification was based on comparison of the compounds mass spectral data (MS) and Kovats retention indices (RI) with those of NIST Mass Spectral Library (2011), Wiley Registry of Mass Spectral Data 8th edition and literature

### Cytotoxicity assay

**Fig. 2:**
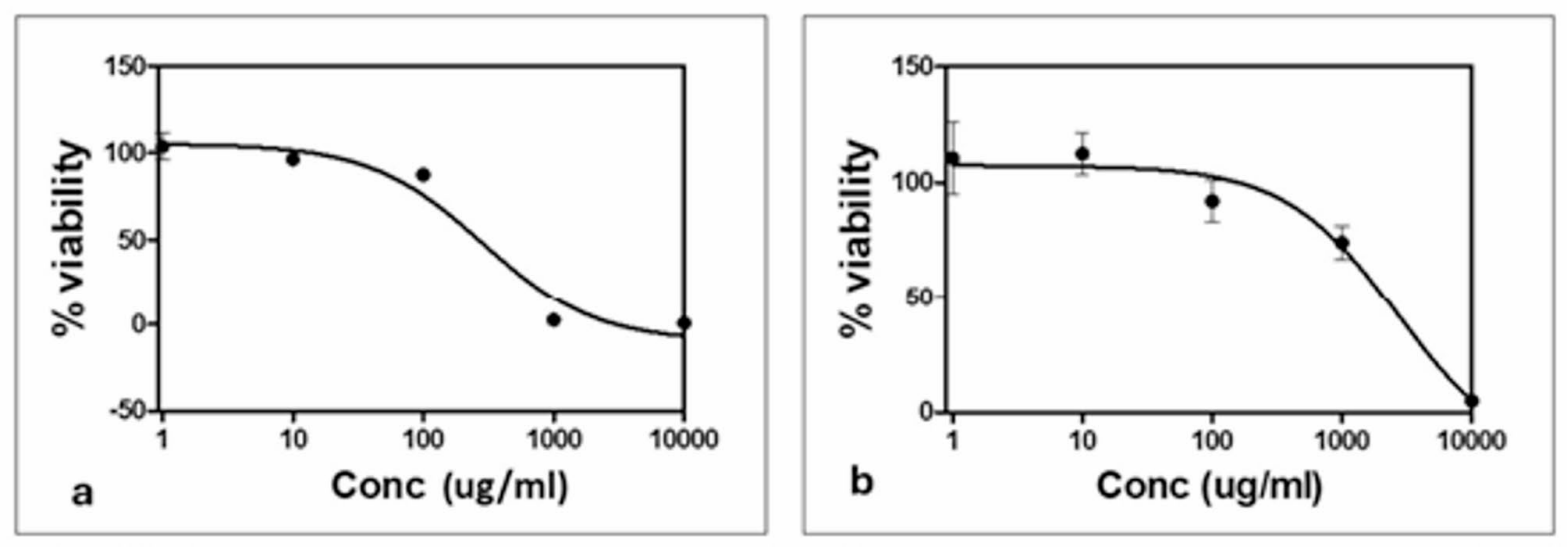
Plot of Percentage cell viability against concentration of tested oil used to calculate IC50. **a**: Caco-2 cell viability against concentration of tested oil, **b**: Fibroblast HFB4 cell viability against concentration of tested oil

**Fig. 3:**
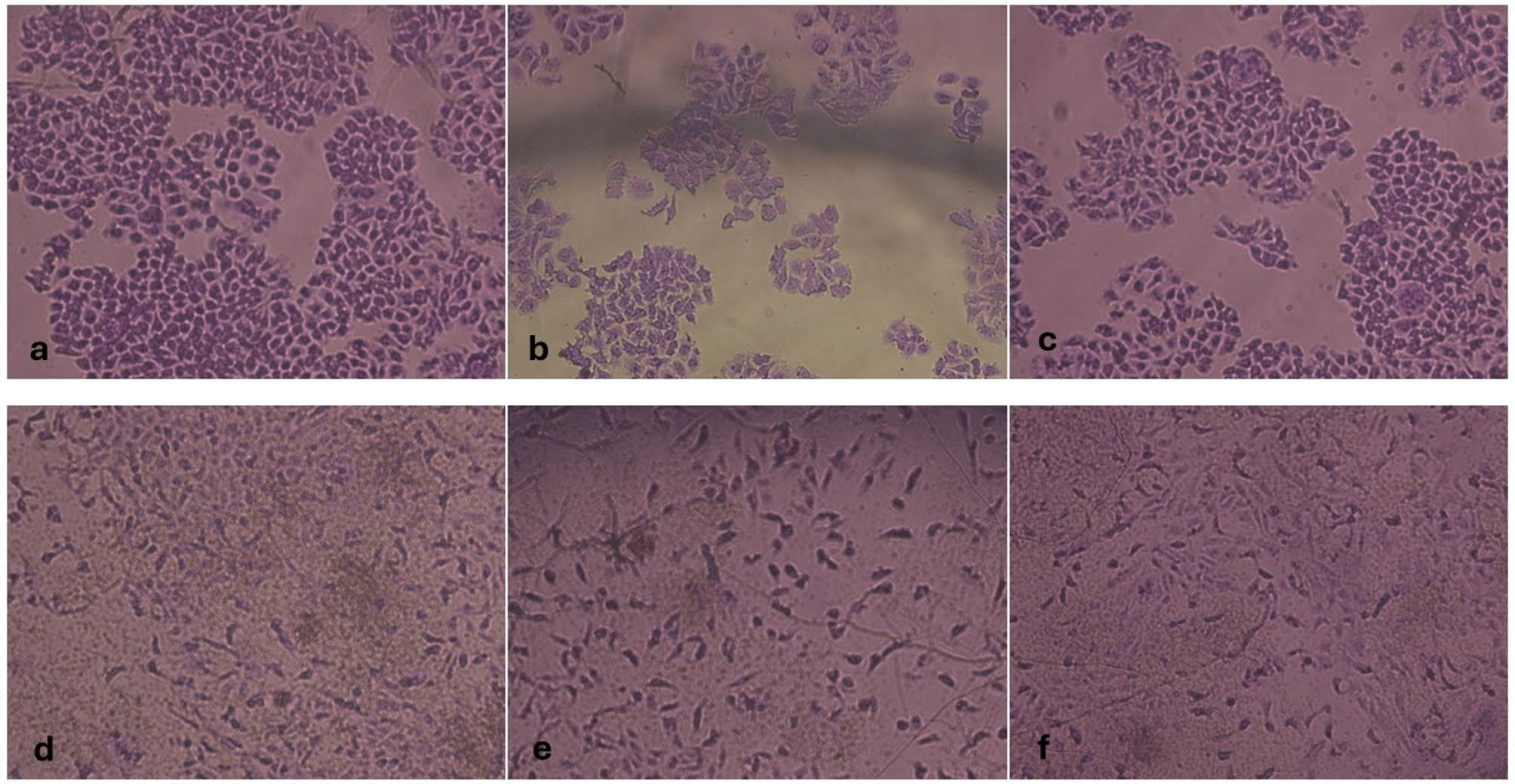
Images of cytotoxicity assays showing Caco-2 and Fibroblast HFB4 cell lines under inverted microscope. **a**: Caco-2 cell line control, **b**: Caco-2 cell line treated with oil at concentration 1000 µg /mL. **c**: Caco-2 cell line treated with oil at concentration 10 µg /mL. **d**: Fibroblast HFB4 cell line control, **e**: Fibroblast HFB4 treated with oil at concentration 1000 µg /mL. **f**: Fibroblast HFB4 line treated with oil at concentration 10 µg /mL

Results showed that for Caco-2 cell line (Fig. 2), essential oil of the leaves showed an IC_50_ value of 277.36 ± 10.14 µg/mL, which is higher than the control drug doxorubicin (1.88 ± 0.274 µg/mL). When tested against fibroblast (HFB4), the IC_50_ value was found to exceed 1000 µg/mL suggesting complete safety and cytocompatibility of the tested oil. In addition, no alterations in cell morphology were observed in the treated cells compared to the untreated controls (Fig. 3).

### In silico molecular docking studies

**Fig. 4:**
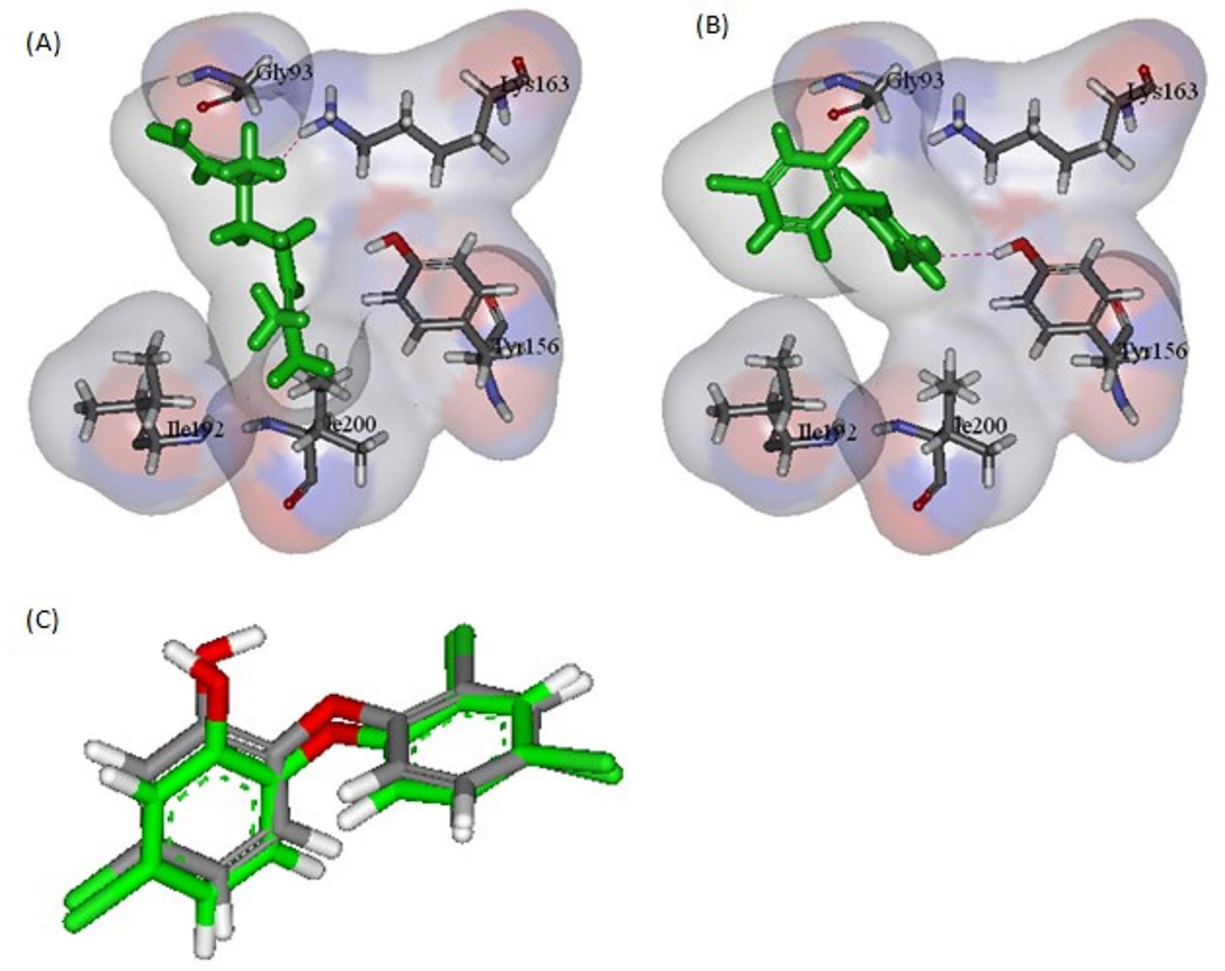
(**A**) and (**B**) represent binding of Linalool and Triclosan in FabI active site, respectively. (**C**) Validation of the docking procedure through RMSD investigation

Interpretation of the -CDOCKER interaction energy revealed that the docking energies of all compounds were comparable to the co-crystallized ligands of FabI and PBP2a of MRSA, and shikimate kinase of *A. baumannii*. For MRSA targets, FabI and PBP2a, the energies ranged from − 18.87 to -26.79 Kcal/mol and − 20.31 to -28.54 Kcal/mol, respectively. Both linalool and α-terpineol revealed the highest binding affinities on both targets, where their binding energies were − 26.79 and − 24.15 Kcal/mol towards FabI, respectively (Table 3). This is consistent with the co-crystallized antimicrobial drug triclosan which displayed interaction energy of -31.32 Kcal/mol. Linalool demonstrated similar binding mode to triclosan where H-bond is formed with Lys163 within its active site (Fig. 4).

**Table 3:** -CDOCKER interaction energies (Kcal/mol) of the major *M. tangelo* essential oil constituents on the selected targets

Compound name	-CDOCKER interaction energies (Kcal/mol)		
	MRSA		A. baumannii
	FabI	PBP2a	Shikimate kinase
*β*-Pinene	20.82	20.40	24.23
*β*-Myrcene	20.54	21.67	23.57
*o*-Cymene	19.79	23.09	21.32
Linalool	**26.79**	**26.58**	**30.61**
Isoterpinoline	18.94	23.43	20.59
*γ*-Terpinene	19.66	21.89	20.86
D-Limonene	18.98	21.88	23.80
*cis*-8-Ocimene	20.56	21.29	19.48
*α*-Thujene	21.16	22.78	25.8
*α*-Terpineol	**24.15**	**28.54**	25.56
*α*-Pinene	18.87	20.49	23.71
Mineola	21.32	20.31	23.09
Co-crystallized ligand	31.32	45.50	37.16
RMSD (A^o^)	0.55	1.173	1.88

**Fig. 5:**
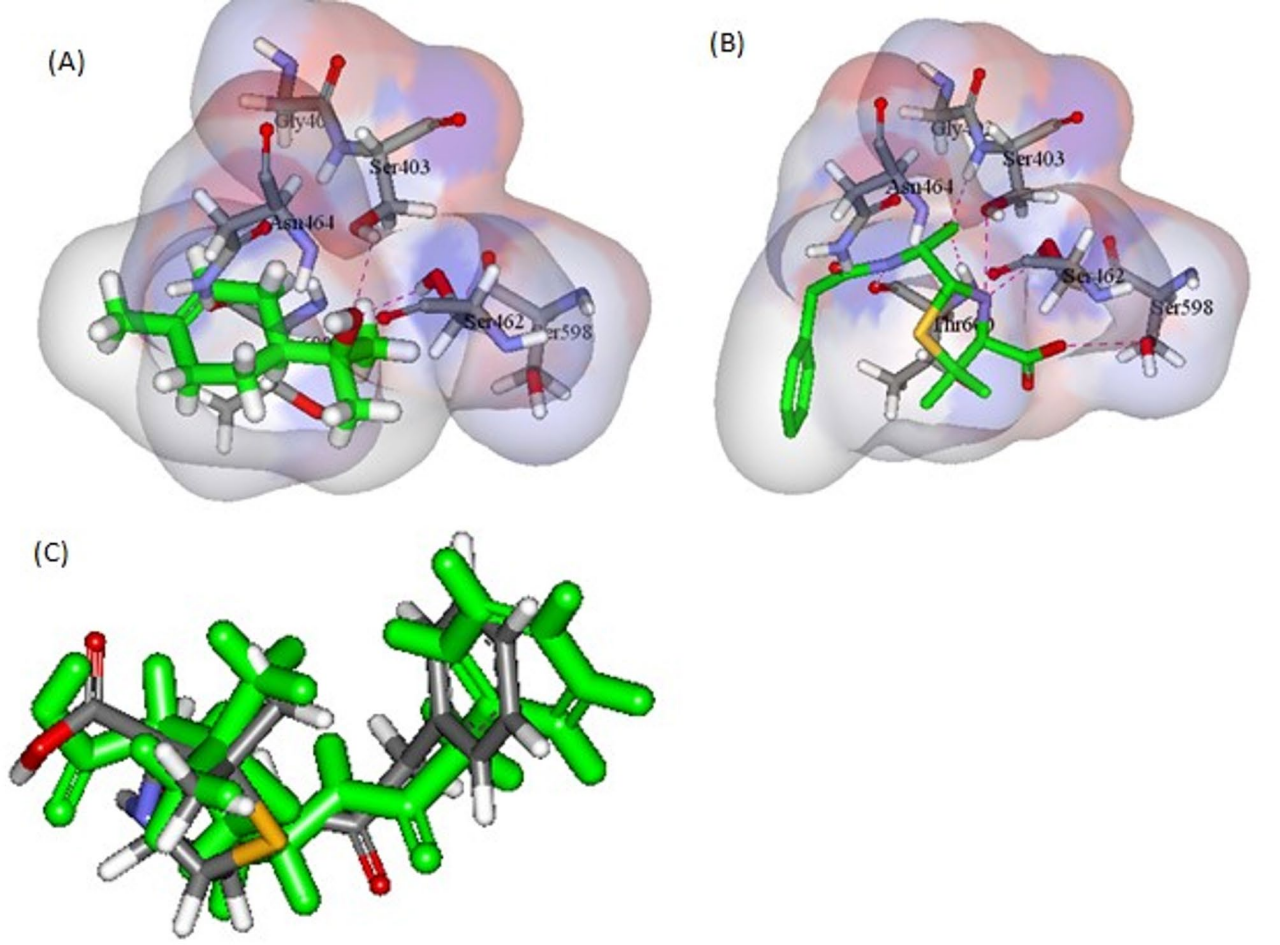
(**A**) and (**B**) demonstrate α-terpineol and penicillin G interactions within the active site of PBP2a, respectively. (**C**) RMSD validation of the docking results

Both compounds, linalool and α-terpineol, also showed the best interaction energies with PBP2a (-26.58 and − 28.54 Kcal/mol, respectively). α-Terpineol displayed higher binding affinity with the manifestation of the two formed hydrogen bonds with Ser403 and Ser462 (Fig. 5).

**Fig. 6:**
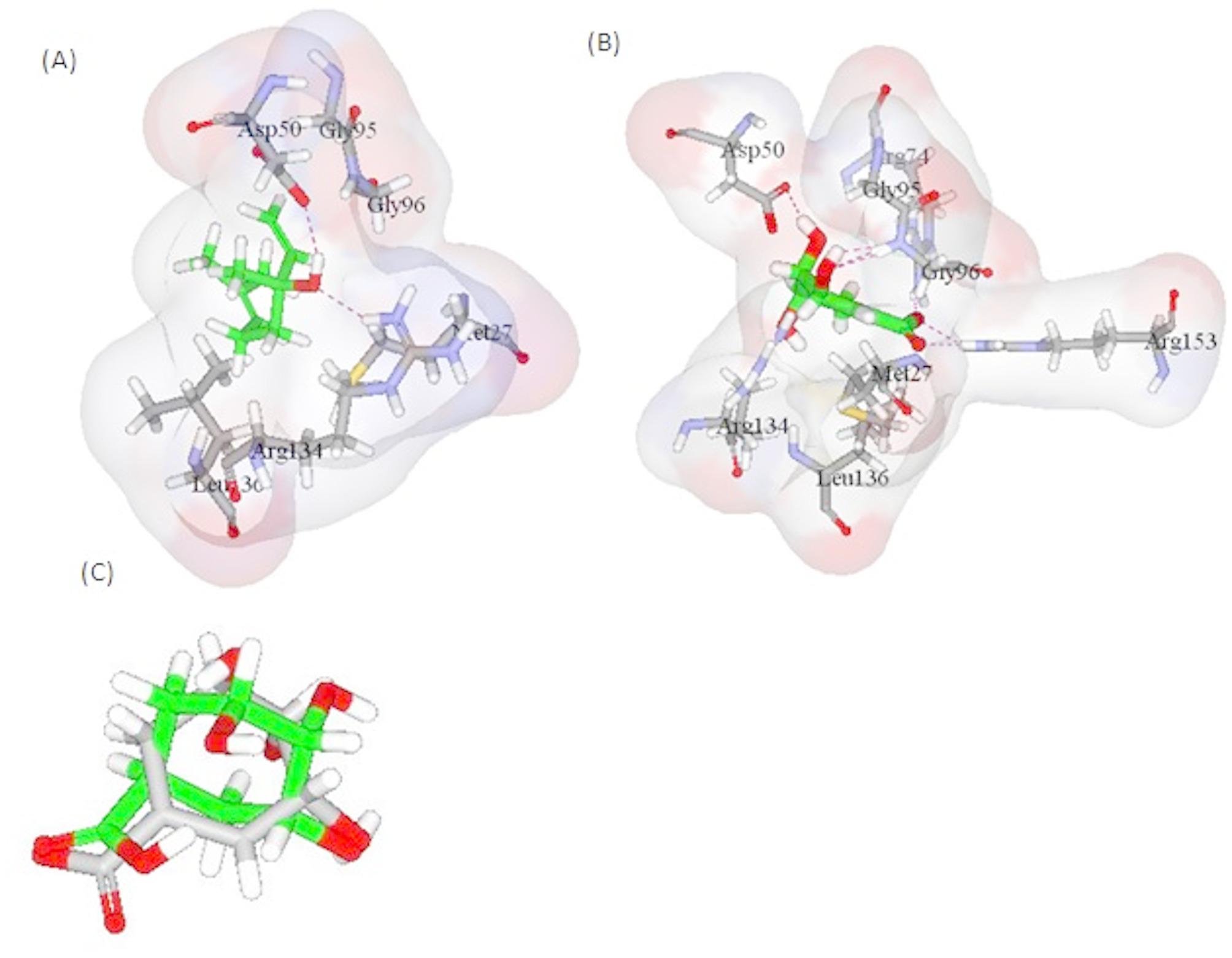
Linalool and shikimate binding in shikimate kinase binding site are presented in (**A**) and (**B**), respectively. (**C**) RMSD of co-crystallized shikimate along with docked pose to reveal the validity of the results

For *A. baumannii* targets, all major constituents were of appreciable -CDOCKER interaction energy upon binding with shikimate kinase as the values were ranging from 19.48 to 30.61 Kcal/mol with linalool revealing the best interaction with the enzyme compared to the co-crystallized ligand: shikimate (30.61 Kcal/mol for linalool and 37.16 Kcal/mol for shikimate) (Table 3). It was found that linalool kept the essential H-bond interaction with Asp50 in the active site with an additional H-bond formed with Arg134 (Fig. 6). Noteworthy, the RMSD values; calculated according to the superimposition of the co-crystalized ligands with their corresponding docked pose, were demonstrated to be < 2 A^o^ which indicated the validity of the docking results.

## Discussion

Multidrug-resistant pathogens pose a significant threat to the public health expressed by their ability to resist the effects of multiple antibiotics. Among the most concerning are *A. baumannii.* and MRSA. These bacteria have developed resistance mechanisms that render common antibiotics ineffective, leading to increased morbidity and mortality rates both globally and in Egypt [14, 15]. Alternative approaches, like the use of essential oils, have therefore gained attention as potential solutions that can either be used alone or in combination with antibiotics [16]. The complex chemical composition of essential oils and diverse modes of action can help overcome bacterial resistance. Furthermore, essential oils have been extensively studied for their cytotoxic activity against cancer cell lines [17]. In this study, we aimed to explore the antibacterial and cytotoxic activity of *M. tangelo* essential oil obtained from the leaves and fruit peels.

Clinical isolates of *A. baumannii* and MRSA were collected from Al Demerdash hospital. Antibiogram analysis of the collected isolates revealed extensive resistance against the tested antibiotics. Vancomycin was the only tested antibiotic active against *A. baumannii* isolates. The three tested isolates were resistant to imipenem belonging to the carbapenem class of antibiotics. Carbapenems are supposed to be the last resort agents for treating resistant *A. baumannii* infections. This confers with the finding of a previous study which reported that the emergence of carbapenem resistance among *A. baumannii* in Egypt is increasing at an alarming rate [15]. Two of the tested MRSA isolates were sensitive only to Linezolid and one isolate was sensitive to both Vancomycin and Linezolid.

Essential oils of both leaves and fruit peel of *M. tangelo* (Tangelo) cultivated in Egypt were obtained by hydrodistillation. Upon testing the antibacterial activity of the essential oil of the fruit peel extract, no notable activity was observed while the essential oil of the leaves displayed a promising activity against tested MRSA and *A. baumannii* isolates.

Chemical profiling of the essential oils from the leaves and fruit peels showed differences in their chemical composition, which justify the variations in their antibacterial activity. Only one report was traced on the leaf essential oil of tangelo cultivated in Greece, which identified limonene (32.25%), 1,8-cineole (10.31%), and linalool (2.31%) as the major constituent [18]. While previous reports on fruit peel oil consistently highlight the predominance of limonene though its percentage varies. Additionally, the composition of other constituents differs across samples from different countries. For instance, Mitiku et al. reported that the fruit peels of the Ethiopian Orlando tangelo and the Kenyan *M. tangelo* hybrids, showed high predominance of limonene (96% and 91% of the cold pressed oils, respectively) followed by myrcene which averaged between 1.7 and 1.9% [19]. Another study showed that Minneola peel is dominant with limonene (90%), followed by γ-terpinene (2.9%), terpinolene (1.7%), and then myrcene (1.5%) [20]. Goldenberg et al. showed the predominance of limonene in Tangelo followed by linalool then myrcene [21]. In our study, peels-derived oils showed predominance of linalool (31.6%) followed by *cis*-β-ocimene (16.1%), while the fruit peel oil showed up to 82% predominance of D-limonene followed by γ-terpinene (5.13%).

Essential oils have also been extensively studied for their cytotoxic activity against various cancer cell lines; numerous studies have demonstrated the potential anticancer effects of essential oils in inhibiting growth and inducing apoptosis in tumor cells [22, 23]. Regarding the safety of essential oils on normal cell lines, it is generally recognized that essential oils exhibit a lower toxicity towards normal cells which is advantageous when applying these essential oils as antimicrobial agents. Accordingly, the present study also tested the cytotoxicity of the collected leaf essential oil - which displayed good antibacterial activity against the tested clinical isolates - against both cancer (Caco-2) and normal (HFB4) cell lines. The results revealed weak cytotoxic activity against Caco-2 cells. However, given that the cytotoxic effects of essential oils can vary across different cell lines, further investigations using additional cancer cell lines are necessary to fully assess any potential cytotoxicity. On the other hand, the tested essential oil exhibited no cytotoxicity against HFB4 cells, highlighting its promising safety profile. This is particularly important, as cytocompatibility—rather than cytotoxicity—is a key criterion for developing a safe antibacterial agent. Furthermore, no morphological alterations were observed in either cell line, reinforcing the oil’s biocompatibility.

In a trial to explore the antibacterial mechanism of action of the major *M. tangelo* essential oil constituents, a comprehensive molecular docking study was conducted on different molecular targets involving various bacterial mechanisms. For MRSA, Enoyl acyl carrier protein reductase (FabI) was identified as an appealing target that inhibits fatty acid biosynthesis in different pathogens including MRSA [24] where natural flavonoid derivatives were confirmed to be potent FabI inhibitors [25]. On the other hand, PBP2a is a substantial enzyme in bacterial cell wall biosynthesis that gained researchers’ attention towards its inhibition as a potential strategy for developing anti-bacterial agents [26]. Other studied possible targets included tyrosyl-tRNA synthetase, another enzyme which is crucial for protein synthesis in the bacterial cell [27], and topoisomerase II DNA gyrase, responsible for introducing supercoils to DNA and considered essential for DNA replication [28]. Studied possible targets in *A. baumannii* included carbapenemase enzyme responsible for the increasing resistance in this pathogen, DNA gyrase and shikimate kinase which is an essential enzyme with a significant role in the metabolic process [29, 30].

The preliminary docking study for all the previous targets revealed that FabI, PBP2a, and shikimate kinase were the most promising targets. By interpreting the -CDOCKER interaction energies, it was observed that the docking energies of all the compounds were comparable to the co-crystallized ligands which were Triclosan (TCL), Penicillin G (PNM), Phosphoaminophosphonic acid-adenylate ester (ANP), Shikimate (SKM), respectively. Both linalool and α-terpineol revealed the highest binding affinities towards the MRSA targets, FabI and PBP2a. Linalool demonstrated binding to FabI similar to that of triclosan with H-bond formed with Lys163 within its active site (Fig. 4). *α*-terpineol was of higher binding affinity to PBP2a with the manifestation of two hydrogen bonds with Ser403 and Ser462 (Fig. 5). Also, all the major constituents of the tested oil were of appreciable -CDOCKER interaction energy upon binding with shikimate kinase of *A. baumannii*, with linalool revealing the best interaction (Table 3). Linalool kept the essential H-bond interaction with Asp50 in the active site with an additional H-bond formed with Arg134 (Fig. 6). This is consistent with a previous study which reported that linalool, a mjor component of the leaf oil, displayed its antimicrobial activity by interfering with essential enzymes [31]. This finding also confirms the interesting role of shikimate kinase as a target for developing novel antibiotics [30]. This result also explains why the fruit peel oil-with low concentration of linalool-lacks antibacterial activity whereas leaf oil, which contains linalool as a major component, displayed good antibacterial activity.

While this study contributes to the understanding of the efficacy of essential oil of *M. tangelo*, it is important to acknowledge certain limitations that may influence the interpretation of the results. For instance, the molecular structures of some possible targets are not available in the PDB. Accordingly, the possible binding of the major constituents of the studies essential oil with such targets could not be assessed. Also, further investigations into the activity of the essential oil against other MDR pathogens as well as other cell lines are necessary to confirm its activity and ensure its safety. In Vivo studies are also needed to further confirm its therapeutic efficacy.

## Conclusions

Chemical profiling of the oil extracted from the leaves and the peels of Egyptian *M. tangelo* was conducted for the first time in this study. The huge difference in the chemical composition was displayed in their antibacterial activity where leaf essential oil showed good inhibitory activity against multidrug resistant pathogens, MRSA and *A. baumannii* whereas peel oil was found to be inactive against the tested isolates. Activity against MRSA could possibly be due to targeting FabI and PBP2a mediated by linalool and α-terpineol as shown by the docking study, whereas activity against *A. baumannii* could possibly involve interaction of linalool with the enzyme shikimate kinase. With a high safety profile, the essential oil of *M. tangelo* leaf has the potential to be developed into an antibacterial drug against MDR pathogens. Displaying weak cytotoxic activity against Caco-2 cell line, further studies are still required to evaluate its cytotoxicity using other cell lines.

## Electronic supplementary material

Below is the link to the electronic supplementary material.

Supplementary Material 1

## Data Availability

No datasets were generated or analysed during the current study.

## Abbreviations

AMR: Antimicrobial-resistance
Caco-2: Colon cancer cell line
DMEM media: Dulbecco’s Modified Eagle Medium
FabI: Enoyl-acyl carrier protein reductase
GC-MS: Gas chromatography coupled to mass spectrometry
HFB4: Fibroblast cell line
KI: Kovats retention index
MHA plates: Mueller-Hinton Agar plates
MIC: Minimum inhibitory concentration
MRSA: Methicillin-resistant Staphylococcus aureus
NIST: National Institute of Standards and Technology
PBP2a: Penicillin binding protein
RMSD: Root Mean Square Deviation
RPMI 1640 media: Roswell Park Memorial Institute 1640 medium
SD: Standard Deviation
SRB: sulforhodamine B
t_R_: Retention Time
PDB: Protein Data Bank
