# Supplementary material for: Minneola tangelo essential oil exhibits antibacterial activity against multidrug-resistant pathogens while maintaining cell safety
Source: BMC Complement Med Ther. 2025 Jul 22;25:281. doi: 10.1186/s12906-025-05015-5 (PMC12281854; doi:10.1186/s12906-025-05015-5)
Supplement: Supplementary file 1 — Supplementary Material 1 [file 12906_2025_5015_MOESM1_ESM.docx]

**Table S1. Resistance profile of the tested MRSA and *A. baumannii* isolates**

| **Antibiotic tested** | **MRSA 1** | **MRSA 2** | **MRSA 3** | **MRSA 4** | **Acin 1** | **Acin 2** | **Acin 3** |
| --- | --- | --- | --- | --- | --- | --- | --- |
| Azithromycin (AZM) 15 µg | R | R | R | S | NA* | NA | NA |
| Cefoxitin (FOX) 30 µg | R | R | R | R | NA | NA | NA |
| Vancomycin (VA) 30 µg | R | S | R | S | S | S | S |
| Linezolid (LZD) 30 µg | S | R | S | R | NA | NA | NA |
| Doxycycline (DO) 30 µg | R | S | R | S | NA | NA | NA |
| SXT 25 µg | R | R | R | S | R | R | R |
| Imipenem (IPM) 10 µg | NA | NA | NA | NA | R | R | R |
| Ceftriaxone (CRO) 30 µg | NA | NA | NA | NA | R | R | R |
| Tetracycline (TGC) 15 µg | NA | NA | NA | NA | R | R | R |
| Gentamycin (CN) 10 µg | NA | NA | NA | NA | R | R | R |

NA *: not assessed.
